# Supplementary figures and images for: Predictive factors requiring high-dose evocalcet in hemodialysis patients with secondary hyperparathyroidism
Source: PLoS One. 2022 Dec 13;17(12):e0279078. doi: 10.1371/journal.pone.0279078 (PMC9746983; doi:10.1371/journal.pone.0279078)

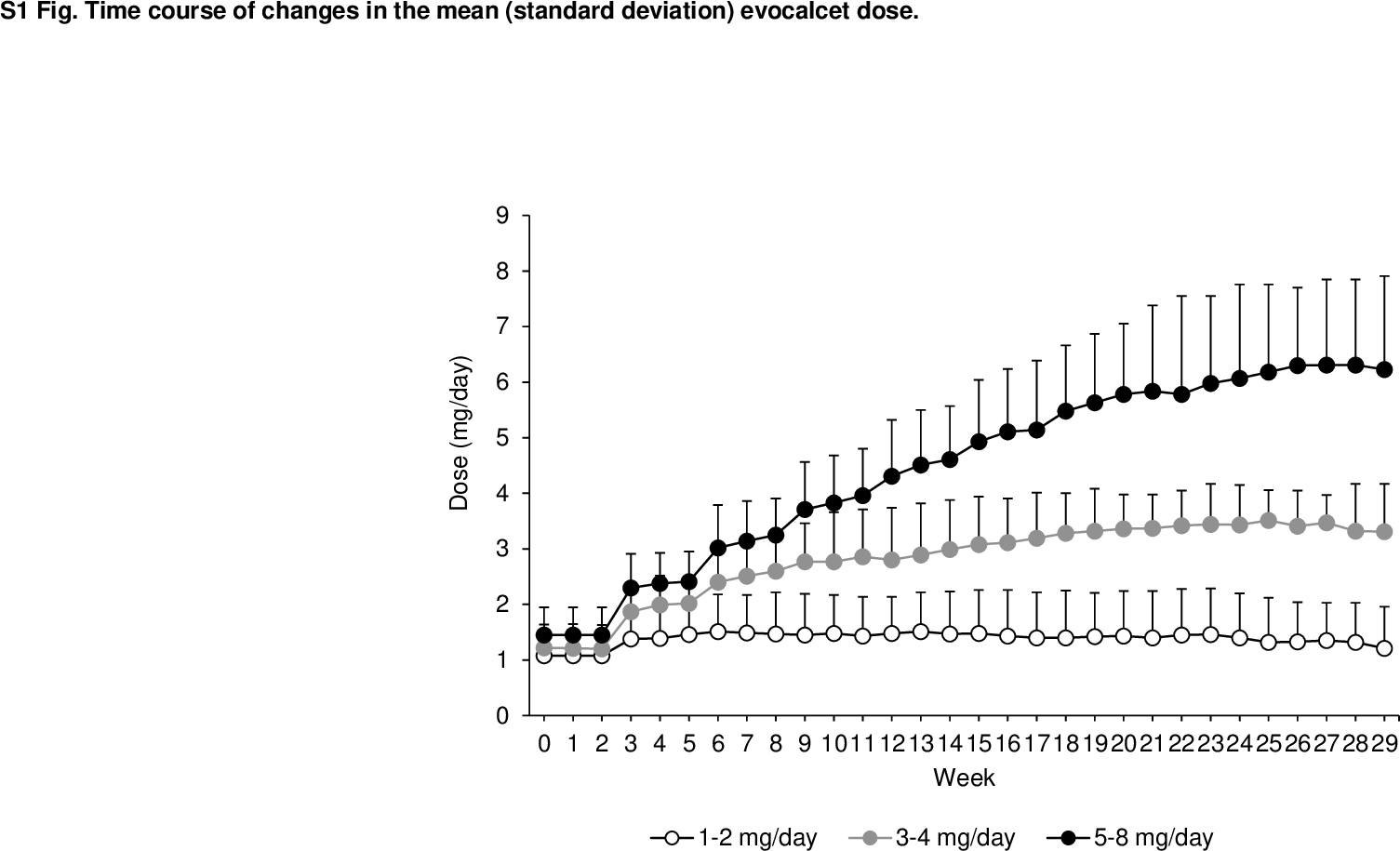

Supplement: S1 Fig — (TIF) [file pone.0279078.s001.tif]
