# Supplementary material for: Predictive factors requiring high-dose evocalcet in hemodialysis patients with secondary hyperparathyroidism
Source: PLoS One. 2022 Dec 13;17(12):e0279078. doi: 10.1371/journal.pone.0279078 (PMC9746983; doi:10.1371/journal.pone.0279078)
Supplement: S1 Table — (PDF) [file pone.0279078.s002.pdf]

S1 Table. List of Institutional Review Boards

Medical Institutions	Name of Institutional Review Board
Asahikawa-Kosei General Hospital	Asahikawa-Kosei General Hospital IRB
Nikko Memorial Hospital	Nikko Memorial Hospital IRB
Sapporo Hokuyu Hospital	Sapporo Medical Association's IRB
Higashi-Naebo Hospital	Sapporo Medical Association's IRB
H • N • MEDIC	Sapporo Medical Association's IRB
H • N • MEDIC Kita-Hiroshima	Sapporo Medical Association's IRB
H • N • MEDIC Sapporo Higashi	Sapporo Medical Association's IRB
Kojinkai Chuou Clinic	Koyasu Neurosurgical Clinic IRB
Kimachi Hospital	Koyasu Neurosurgical Clinic IRB
Rifunonaika Clinic (Miyata Rifu Clinic)	Koyasu Neurosurgical Clinic IRB
Dainohara Clinic	Koyasu Neurosurgical Clinic IRB
Kawadaira Naika Medical Clinic	Koyasu Neurosurgical Clinic IRB
Tokiwakai Joban Hospital	Koyasu Neurosurgical Clinic IRB
Ibaraki Seinan Medical Center Hospital	Review Board of Human Rights and Ethics for Clinical Studies
Tokiwa Clinic	Koyasu Neurosurgical Clinic IRB
Kikuchi Medical Clinic	Koyasu Neurosurgical Clinic IRB
Ohishi Naika Clinic	Tokyo Midtown Clinic IRB
Tsuchiura Beryl Clinic	Koyasu Neurosurgical Clinic IRB
Ora Hospital	Review Board of Human Rights and Ethics for Clinical Studies
Heisei Hidaka Clinic	Review Board of Human Rights and Ethics for Clinical Studies
Kubojima Clinic	Koukeikai Sugiura Clinic IRB
Bosei Hospital	Medical Corporation SHOWAKAI IRB
Hakuyukai Yuai Nisshin Clinic	Hoshikuma Dermatology / Allergy Department Clinic IRB
Oshima Clinic	Koukeikai Sugiura Clinic IRB
Saitama Tsuki no Mori Clinic	Kojinkai Medical Corporation Shin-Nihonbashi Ishii Clinic IRB
Japan Community Health Care Organization Chiba Hospital	Eijyukai Ryohoku Hospital IRB
Asahi General Hospital	Asahi General Hospital IRB
Kohitsujikai Naganuma Clinic	Review Board of Human Rights and Ethics for Clinical Studies
Tokatsu Clinic Mirai	Review Board of Human Rights and Ethics for Clinical Studies
St. Luke’s International Hospital	St.Luke's International Hospital IRB
Toranomon Hospital	Toranomon Hospital and Toranomon Hospital Kajigaya IRB
Shinshikai Hachioji Azumacho Clinic	Koukeikai Sugiura Clinic IRB
Ayase Ekimae Jin Clinic	Medical Corporation SHOWAKAI IRB

Medical Institutions	Name of Institutional Review Board
Futago-tamagawa Ekimae Clinic	Koukeikai Sugiura Clinic IRB
Showakai Bosei Shinjuku Minamiguchi Clinic	Medical Corporation SHOWAKAI IRB
Kita Hachiouji Clinic	Medical Corporation SHOWAKAI IRB
Toshiba Rinkan Hospital	Koyasu Neurosurgical Clinic IRB
Bosei Fujisawa Clinic	Medical Corporation SHOWAKAI IRB
Shinjinkai Yokosuka Clinic	Kojinkai Medical Corporation Shin-Nihonbashi Ishii Clinic IRB
Maeda Institute of Renal Research Musashikosugi Clinic	Koyasu Neurosurgical Clinic IRB
Tachibanadai Hospital	Koukeikai Sugiura Clinic IRB
Sekishinkai Kawasaki Clinic	Koukeikai Sugiura Clinic IRB
Bosei Hiratsuka Clinic, Medical Corporation Showakai	Medical Corporation SHOWAKAI IRB
Boseikai Honatsugi Medical Clinic	Medical Corporation SHOWAKAI IRB
Koshikawakinen Yokohama Jin Clinic	Review Board of Human Rights and Ethics for Clinical Studies
Shinjinkai Kurihama Clinic	Kojinkai Medical Corporation Shin-Nihonbashi Ishii Clinic IRB
Maeda Institute of Renal Research Shin-Yokohama Clinic	Koyasu Neurosurgical Clinic IRB
Niigata City Shakai Jigyo Kyokai Shinrakuen Hospital	Niigata City Shakai Jigyo Kyokai Shinrakuen Hospital IRB
Fujikoshi Hospital	Kojinkai Medical Corporation Shin-Nihonbashi Ishii Clinic IRB
Ueda Jinzo Clinic	Review Board of Human Rights and Ethics for Clinical Studies
Japanese Red Cross Society Suwa Hospital	Review Board of Human Rights and Ethics for Clinical Studies
Aizawa Hospital	Aizawa Hospital IRB
Ueda Tohseki Clinic	Review Board of Human Rights and Ethics for Clinical Studies
Kanno Dialysis & Vascular Access Clinic	Review Board of Human Rights and Ethics for Clinical Studies
Miyaji Clinic	Kojinkai Medical Corporation Shin-Nihonbashi Ishii Clinic IRB
Fuji Daiichi Clinic	Medical Corporation SHOWAKAI IRB
Fuyo Association Seirei Numazu	Fuyo Association Seirei Numazu IRB
Masuko Clinic Subaru	Masuko Memorial Hospital IRB
Masuko Hospital	Masuko Memorial Hospital IRB
Daiyukai Daiichi Hospital	Daiyukai Daiichi Hospital IRB
Meiyo Clinic	Review Board of Human Rights and Ethics for Clinical Studies
Sanen Medi Mates Toyohashi Mates Clinic	Sanen Medi Mates Toyohashi Mates Clinic Sleep Center IRB

**Medical Institutions**	**Name of Institutional Review Board**
Anjyo Kyoritsu Clinic	Kaikoukai Healthcare Corporation Nagoya Kyoritsu Hospital IRB
Takeuchi Hospital	Kondo Hospital IRB
Nishijin Hospital	Koyasu Neurosurgical Clinic IRB
Tojinkai Hospital	Kojinkai Medical Corporation Shin-Nihonbashi Ishii Clinic IRB
Rakuwakai Otowa Memorial Hospital	Rakuwakai Otowa Hospital IRB
Kyotoekimae Takeda Tohseki Clinic	Koseikai Takeda Hospital Group IRB
Fuchu Hospital	Koukeikai Sugiura Clinic IRB
Soryukai Inoue Hospital	Soryukai Inoue Hospital IRB
General Hospital Higashikouri	Review Board of Human Rights and Ethics for Clinical Studies
Nozaki Clinic	Koukeikai Sugiura Clinic IRB
Shirasagi Minami Hospital	Review Board of Human Rights and Ethics for Clinical Studies
Chibune Kidney and Dialysis Clinic, Aijinkai Chibune General Hospital	Aijinkai Chibune General Hospital IRB
Shigei Medical Research Hospital	Shigei Medical Research Hospital IRB
Sowakai Shigei Hospital	Kojinkai Medical Corporation Shin-Nihonbashi Ishii Clinic IRB
Chuo Naika Clinic	Koyasu Neurosurgical Clinic IRB
Ichiyokai Clinic	Koukeikai Sugiura Clinic IRB
Nakajima Tsujiya Clinic	Akane Foundation Tsuchiya General Hospital IRB
Saint Hill Hospital	Koukeikai Sugiura Clinic IRB
Tokuyama Central Hospital	Tokuyama Central Hospital IRB
Kawashima Tohseki Clinic	Social medical corporation Kawashima society Kawashima Hospital IRB
Kinashi Obayashi Hospital	Koukeikai Sugiura Clinic IRB
Takayama Hospital	Hoshikuma Hifuka Allergy Clinic IRB
Shin Koga Hospital	Shin Koga Hospital IRB
Nagasaki Jin Hospital	Tokyo Midtown Clinic IRB
Matsushitakai Akebono Clinic	Kojinkai Medical Corporation Shin-Nihonbashi Ishii Clinic IRB
Seijinkai Ikeda Hospital	Seijinkai Ikeda Hospital IRB
Tomishiro Central Hospital	Koyasu Neurosurgical Clinic IRB
