# Supplementary material for: Predictive factors requiring high-dose evocalcet in hemodialysis patients with secondary hyperparathyroidism
Source: PLoS One. 2022 Dec 13;17(12):e0279078. doi: 10.1371/journal.pone.0279078 (PMC9746983; doi:10.1371/journal.pone.0279078)
Supplement: S2 Table — (PDF) [file pone.0279078.s003.pdf]

S2 Table. Incidence of major adverse drug reactions

	Final evocalcet dosages (mg/day)			
	1-2	3-4	5-8	Total
Week 28 to week 30, n	101	75	83	259
Hypocalcemia-related adverse drug reactions, n (%)	1 (1.0)	0 (0)	2 (2.4)	3 (1.2)
Gastrointestinal tract-related adverse drug reactions, n (%)	0 (0)	1 (1.3)	0 (0)	1 (0.4)
Decreased appetite	0 (0)	1 (1.3)	0 (0)	1 (0.4)
Week 0 to week 28, n	135	90	92	317
Hypocalcemia-related adverse drug reactions, n (%)	33 (24)	11 (12)	13 (14)	57 (18)
Gastrointestinal tract-related adverse drug reactions, n (%)	17 (13)	13 (14)	11 (12)	41 (13)
Nausea	8 (6)	6 (7)	2 (2)	16 (5)
Vomiting	4 (3)	4 (4)	6 (7)	14 (4)
Abdominal discomfort	5 (4)	3 (3)	2 (2)	10 (3)
Abdominal distension	1 (1)	0 (0)	1 (1)	2 (1)
Decreased appetite	3 (2)	2 (2)	2 (2)	7 (2)
