# Supplementary material for: Predictive factors requiring high-dose evocalcet in hemodialysis patients with secondary hyperparathyroidism
Source: PLoS One. 2022 Dec 13;17(12):e0279078. doi: 10.1371/journal.pone.0279078 (PMC9746983; doi:10.1371/journal.pone.0279078)
Supplement: S3 Table — (PDF) [file pone.0279078.s004.pdf]

S3 Table. Univariate analyses of factors associated with medium- and/or high final evocalcet dosages using patient pre-treatment characteristics as variables

Variables	Final evocalcet dosages	Univariate analysis		
		OR	95% CI	*p*-value
Sex (male)	Medium	1.17	0.64–2.12	0.62
	High	0.92	0.52–1.64	0.78
Age (year)	Medium	0.95	0.93–0.98	<0.001
	High	0.95	0.92–0.97	<0.001
Primary kidney disease (DN)	Medium	0.54	0.30–1.00	0.05
	High	0.41	0.22–0.77	0.005
Duration of dialysis (year)	Medium	1.06	1.02–1.11	0.004
	High	1.10	1.06–1.15	<0.001
Type of dialysis (HD)	Medium	0.68	0.33–1.38	0.28
	High	0.37	0.19–0.71	0.003
Dialysis efficiency (per 1)	Medium	1.51	0.60–3.82	0.38
	High	1.97	0.78–4.94	0.15
Prior cinacalcet use	Medium	2.71	1.56–4.72	<0.001
	High	8.74	4.48–17.05	<0.001
Intravenous VDRA use	Medium	0.51	0.30–0.88	0.016
	High	0.41	0.24–0.71	0.002
P binder use (number of types)	Medium	1.22	0.88–1.70	0.24
	High	1.49	1.06–2.07	0.02
Serum iPTH level (log)	Medium	119.91	12.16–>1000.00	<0.001
	High	>1000.00	367.83–>1000.00	<0.001
Serum corrected Ca level (mg/dL)	Medium	3.01	1.68–5.38	<0.001
	High	8.67	4.64–16.21	<0.001

Serum P level (mg/dL)	Medium	1.11	0.91–1.37	0.30
	High	1.04	0.85–1.28	0.72
Serum P1NP level (log)	Medium	2.13	0.80–5.65	0.13
	High	15.75	5.41–45.80	<0.001
Serum iFGF23 level (log)	Medium	1.80	1.10–2.97	0.02
	High	3.31	1.91–5.74	<0.001
Maximum PTG volume (≥200 mm^3)	Medium	2.45	1.16–5.14	0.018
	High	6.44	3.05–13.59	<0.001

A logistic regression model was applied to assess the week 0 characteristics of patients who required the medium-dose or high-dose range of evocalcet at week 28. Evocalcet dose range: medium, 3–4 mg/day and high, 5–8 mg/day. Ca, calcium; CI, confidence interval; DN, diabetic nephropathy; HD, hemodialysis; iFGF23, intact fibroblast growth factor 23; iPTH, intact parathyroid hormone; OR, odds ratio; P, phosphate; P1NP, procollagen type 1 N-terminal propeptide; PTG, parathyroid gland; VDRA, vitamin D receptor activators.
