# Supplementary material for: Predictive factors requiring high-dose evocalcet in hemodialysis patients with secondary hyperparathyroidism
Source: PLoS One. 2022 Dec 13;17(12):e0279078. doi: 10.1371/journal.pone.0279078 (PMC9746983; doi:10.1371/journal.pone.0279078)
Supplement: S1 File — (PDF) [file pone.0279078.s005.pdf]

S1 File. Serum iPTH, corrected Ca and P levels in patients with SHPT treated with evocalcet for 30 weeks.

		Final evocalcet dosages (mg/day)		
	Week	1-2 (n=131)	3-4 (n=90)	5-8 (n=92)
iPTH (pg/mL),	0	311 (272, 393)	382 (314, 458)	458 (367, 596)
Median (IQR)	1	260 (187, 340)	348 (275, 457)	428 (337, 564)
	2	255 (209, 343)	322 (258, 438)	412 (358, 607)
	3	263 (201, 363)	332 (278, 436)	431 (358, 547)
	4	249 (177, 314)	303 (239, 406)	424 (322, 543)
	5	240 (166, 303)	321 (238, 407)	417 (326, 545)
	6	211 (151, 314)	301 (232, 402)	404 (288, 543)
	7	201 (139, 289)	254 (216, 343)	369 (277, 527)
	8	197 (142, 265)	273 (183, 350)	391 (307, 534)
	9	186 (142, 287)	245 (189, 341)	398 (311, 536)
	10	185 (131, 275)	223 (176, 311)	354 (273, 465)
	11	182 (125, 275)	235 (187, 300)	341 (291, 488)
	12	200 (127, 271)	230 (179, 298)	347 (284, 482)
	13	172 (123, 255)	211 (172, 274)	333 (264, 417)
	14	163 (110, 278)	196 (155, 293)	320 (250, 411)
	15	164 (119, 259)	203 (153, 256)	292 (223, 392)
	16	167 (113, 242)	210 (147, 285)	289 (210, 387)
	17	168 (117, 248)	198 (159, 256)	302 (226, 406)
	18	169 (117, 246)	181 (141, 244)	286 (201, 437)
	19	172 (107, 252)	191 (132, 267)	267 (202, 357)
	20	160 (117, 239)	179 (156, 248)	261 (201, 381)
	21	144 (97, 236)	190 (142, 270)	261 (190, 357)
	22	144 (104, 227)	171 (117, 237)	244 (187, 382)
	23	145 (92, 213)	173 (130, 231)	238 (191, 432)
	24	146 (104, 221)	174 (122, 208)	247 (185, 365)
	25	153 (101, 216)	163 (126, 213)	262 (184, 344)
	26	143 (97, 229)	150 (114, 222)	239 (182, 332)
	27	138 (99, 221)	162 (102, 233)	233 (167, 326)
	28	141 (91, 200)	148 (101, 205)	216 (150, 340)
	29	140 (93, 231)	152 (111, 197)	232 (156, 319)
	30	149 (99, 212)	149 (108, 197)	216 (136, 341)

IQR, interquartile range; iPTH, intact parathyroid hormone; SHPT, secondary hyperparathyroidism

		Final evocalcet dosages (mg/day)		
	Week	1-2 (n=131)	3-4 (n=90)	5-8 (n=92)
Corrected Ca (mg/dL), Mean (SD)	0	9.3 ± 0.5	9.5 ± 0.5	9.8 ± 0.6
	1	8.7 ± 0.5	8.9 ± 0.6	9.2 ± 0.6
	2	8.6 ± 0.5	8.9 ± 0.5	9.2 ± 0.6
	3	8.5 ± 0.5	8.8 ± 0.5	9.1 ± 0.5
	4	8.5 ± 0.6	8.7 ± 0.5	8.9 ± 0.6
	5	8.5 ± 0.5	8.7 ± 0.5	8.9 ± 0.5
	6	8.5 ± 0.6	8.6 ± 0.5	8.9 ± 0.6
	7	8.5 ± 0.6	8.6 ± 0.5	8.8 ± 0.6
	8	8.5 ± 0.6	8.5 ± 0.5	8.7 ± 0.5
	9	8.5 ± 0.6	8.5 ± 0.5	8.7 ± 0.6
	10	8.6 ± 0.7	8.5 ± 0.5	8.6 ± 0.5
	11	8.6 ± 0.6	8.5 ± 0.6	8.7 ± 0.5
	12	8.6 ± 0.6	8.5 ± 0.5	8.7 ± 0.6
	13	8.6 ± 0.6	8.5 ± 0.7	8.6 ± 0.6
	14	8.7 ± 0.7	8.6 ± 0.6	8.7 ± 0.6
	15	8.6 ± 0.7	8.6 ± 0.6	8.6 ± 0.5
	16	8.8 ± 0.7	8.7 ± 0.6	8.6 ± 0.6
	17	8.7 ± 0.7	8.6 ± 0.6	8.6 ± 0.5
	18	8.7 ± 0.7	8.6 ± 0.6	8.6 ± 0.6
	19	8.7 ± 0.7	8.6 ± 0.5	8.6 ± 0.6
	20	8.7 ± 0.7	8.6 ± 0.6	8.6 ± 0.6
	21	8.8 ± 0.7	8.6 ± 0.6	8.5 ± 0.5
	22	8.8 ± 0.7	8.7 ± 0.7	8.6 ± 0.5
	23	8.7 ± 1.1	8.5 ± 0.6	8.6 ± 0.6
	24	8.7 ± 0.7	8.6 ± 0.6	8.6 ± 0.5
	25	8.8 ± 0.7	8.7 ± 0.7	8.6 ± 0.6
	26	8.8 ± 0.7	8.7 ± 0.6	8.6 ± 0.6
	27	8.8 ± 0.6	8.7 ± 0.6	8.6 ± 0.5
	28	8.8 ± 0.9	8.7 ± 0.7	8.7 ± 0.6
	29	8.8 ± 0.6	8.8 ± 0.7	8.6 ± 0.6
	30	8.8 ± 0.7	8.7 ± 0.7	8.7 ± 0.6

SD, standard deviation; Ca, calcium

		Final evocalcet dosages (mg/day)		
	Week	1-2 (n=131)	3-4 (n=90)	5-8 (n=92)
P (mg/dL),	0	5.7 ± 1.3	5.9 ± 1.3	5.8 ± 1.4
Mean (SD)	1	5.3 ± 1.2	5.6 ± 1.3	5.6 ± 1.2
	2	5.3 ± 1.1	5.5 ± 1.3	5.5 ± 1.3
	3	5.3 ± 1.3	5.5 ± 1.5	5.6 ± 1.2
	4	5.1 ± 1.2	5.4 ± 1.2	5.5 ± 1.3
	5	5.3 ± 1.2	5.5 ± 1.2	5.4 ± 1.1
	6	5.2 ± 1.2	5.3 ± 1.1	5.5 ± 1.3
	7	5.1 ± 1.2	5.3 ± 1.2	5.4 ± 1.2
	8	5.1 ± 1.3	5.2 ± 1.2	5.5 ± 1.4
	9	5.1 ± 1.2	5.3 ± 1.3	5.5 ± 1.3
	10	5.1 ± 1.1	5.2 ± 1.3	5.3 ± 1.2
	11	5.0 ± 1.3	5.2 ± 1.3	5.2 ± 1.2
	12	5.0 ± 1.3	5.2 ± 1.4	5.2 ± 1.2
	13	5.1 ± 1.3	5.2 ± 1.2	5.1 ± 1.2
	14	5.3 ± 1.5	5.3 ± 1.3	5.2 ± 1.4
	15	5.0 ± 1.3	5.1 ± 1.2	4.9 ± 1.3
	16	5.0 ± 1.3	5.3 ± 1.4	5.0 ± 1.3
	17	5.1 ± 1.3	5.2 ± 1.2	5.0 ± 1.2
	18	5.1 ± 1.2	5.2 ± 1.2	4.9 ± 1.2
	19	5.0 ± 1.2	5.1 ± 1.3	4.9 ± 1.3
	20	5.0 ± 1.2	5.2 ± 1.4	4.9 ± 1.3
	21	5.0 ± 1.2	5.1 ± 1.3	4.8 ± 1.3
	22	5.1 ± 1.3	5.2 ± 1.5	4.9 ± 1.4
	23	5.0 ± 1.2	5.1 ± 1.4	5.1 ± 1.6
	24	5.1 ± 1.5	5.0 ± 1.3	4.9 ± 1.5
	25	5.1 ± 1.4	5.1 ± 1.4	4.9 ± 1.4
	26	5.1 ± 1.2	5.2 ± 1.6	5.0 ± 1.4
	27	5.2 ± 1.3	4.9 ± 1.3	4.9 ± 1.5
	28	5.1 ± 1.3	5.0 ± 1.2	4.9 ± 1.6
	29	4.9 ± 1.3	5.2 ± 1.4	4.9 ± 1.4
	30	5.0 ± 1.4	5.1 ± 1.3	4.8 ± 1.5

SD, standard deviation; P, phosphate
