# Supplementary material for: Predictive factors requiring high-dose evocalcet in hemodialysis patients with secondary hyperparathyroidism
Source: PLoS One. 2022 Dec 13;17(12):e0279078. doi: 10.1371/journal.pone.0279078 (PMC9746983; doi:10.1371/journal.pone.0279078)
Supplement: S2 File — (PDF) [file pone.0279078.s006.pdf]

S2 File. Ratio of patients with serum iPTH, corrected Ca and P levels above, within, and below the target ranges of Japanese Society for Dialysis Therapy for 30 weeks.

	Week	n	Final evocalcet dosages: 1-2 mg/day			
			Low (n)	Achieved (n)	High (n)	Achievement rate (%)
iPTH	0	131	0	5	126	4
	1	130	0	58	72	45
	2	130	0	60	70	46
	3	127	0	53	74	42
	4	127	1	62	64	49
	5	124	2	62	60	50
	6	124	2	74	48	60
	7	121	4	75	42	62
	8	119	3	78	38	66
	9	117	2	78	37	67
	10	116	4	73	39	63
	11	115	3	75	37	65
	12	112	6	68	38	61
	13	111	6	70	35	63
	14	110	2	72	36	65
	15	108	10	63	35	58
	16	107	3	77	27	72
	17	106	1	77	28	73
	18	106	4	74	28	70
	19	106	5	72	29	68
	20	105	5	74	26	70
	21	101	3	73	25	72
	22	102	3	79	20	77
	23	102	5	78	19	77
	24	102	4	78	20	77
	25	101	4	75	22	74
	26	101	5	76	20	75
	27	101	6	71	24	70
	28	101	10	70	21	69
	29	101	1	78	22	77
	30	99	1	78	20	79

	Week	n	Final evocalcet dosages: 3-4 mg/day			
			Low (n)	Achieved (n)	High (n)	Achievement rate (%)
iPTH	0	90	0	1	89	1
	1	88	0	8	80	9
	2	90	0	17	73	19
	3	90	0	10	80	11
	4	89	0	24	65	27
	5	87	0	24	63	28
	6	87	0	23	64	26
	7	88	0	32	56	36
	8	88	0	34	54	39
	9	86	0	41	45	48
	10	85	0	48	37	57
	11	83	0	44	39	53
	12	83	0	48	35	58
	13	81	0	54	27	67
	14	81	0	52	29	64
	15	79	1	56	22	71
	16	78	1	51	26	65
	17	79	0	54	25	68
	18	77	0	57	20	74
	19	78	1	53	24	68
	20	78	0	54	24	69
	21	77	0	53	24	69
	22	78	0	59	19	76
	23	78	0	60	18	77
	24	76	0	63	13	83
	25	76	3	61	12	80
	26	75	1	59	15	79
	27	74	1	56	17	76
	28	73	1	59	13	81
	29	73	2	56	15	77
	30	73	4	53	16	73
	Week	n	Final evocalcet dosages: 5-8 mg/day			

			Low (n)	Achieved (n)	High (n)	Achievement rate (%)
iPTH	0	92	0	0	92	0
	1	92	0	6	86	7
	2	92	0	5	87	5
	3	91	0	5	86	6
	4	92	0	10	82	11
	5	91	0	8	83	9
	6	92	0	8	84	9
	7	92	0	14	78	15
	8	92	0	13	79	14
	9	91	0	13	78	14
	10	92	0	15	77	16
	11	90	0	10	80	11
	12	90	0	12	78	13
	13	90	0	19	71	21
	14	90	0	19	71	21
	15	90	0	31	59	34
	16	90	0	31	59	34
	17	89	0	29	60	33
	18	90	0	34	56	38
	19	90	0	31	59	34
	20	90	0	41	49	46
	21	90	0	36	54	40
	22	90	0	44	46	49
	23	88	0	45	43	51
	24	84	0	39	45	46
	25	83	0	37	46	45
	26	83	0	42	41	51
	27	82	0	43	39	52
	28	83	0	48	35	58
	29	81	0	41	40	51
	30	81	0	47	34	58

iPTH, intact parathyroid hormone

	Week	n	Final evocalcet dosages: 1-2 mg/day			
			Low (n)	Achieved (n)	High (n)	Achievement rate (%)
cCa	0	131	2	123	6	94
	1	130	33	96	1	74
	2	129	46	82	1	64
	3	126	49	76	1	60
	4	127	60	66	1	52
	5	124	60	63	1	51
	6	124	58	63	3	51
	7	121	58	60	3	50
	8	119	53	64	2	54
	9	117	52	63	2	54
	10	116	44	68	4	59
	11	115	45	67	3	58
	12	112	42	68	2	61
	13	111	46	63	2	57
	14	110	38	67	5	61
	15	108	45	58	5	54
	16	106	28	74	4	70
	17	106	35	67	4	63
	18	106	37	66	3	62
	19	106	40	62	4	58
	20	105	37	62	6	59
	21	101	26	69	6	68
	22	102	31	65	6	64
	23	102	35	58	9	57
	24	102	34	64	4	63
	25	101	28	68	5	67
	26	101	30	65	6	64
	27	101	26	71	4	70
	28	101	33	64	4	63
	29	101	29	67	5	66
	30	99	21	71	7	72
	Week	n	Final evocalcet dosages: 3-4 mg/day			

			Low (n)	Achieved (n)	High (n)	Achievement rate (%)
cCa	0	90	0	76	14	84
	1	88	10	76	2	86
	2	90	14	73	3	81
	3	90	14	74	2	82
	4	89	27	60	2	67
	5	86	22	62	2	72
	6	87	33	53	1	61
	7	88	32	56	0	64
	8	88	37	50	1	57
	9	86	39	47	0	55
	10	85	38	46	1	54
	11	83	34	47	2	57
	12	83	37	45	1	54
	13	81	28	52	1	64
	14	81	30	50	1	62
	15	79	31	48	0	61
	16	78	25	49	4	63
	17	79	29	49	1	62
	18	77	28	46	3	60
	19	78	26	50	2	64
	20	78	33	41	4	53
	21	77	34	40	3	52
	22	78	27	47	4	60
	23	78	36	40	2	51
	24	76	27	46	3	61
	25	76	24	48	4	63
	26	74	20	51	3	69
	27	74	18	53	3	72
	28	73	22	47	4	64
	29	73	24	46	3	63
	30	73	22	46	5	63

	Week	n	Final evocalcet dosages: 5-8 mg/day			
			Low (n)	Achieved (n)	High (n)	Achievement rate (%)

cCa	0	92	0	64	28	70
	1	92	2	84	6	91
	2	92	4	83	5	90
	3	91	2	86	3	95
	4	92	16	72	4	78
	5	91	13	75	3	82
	6	92	19	69	4	75
	7	92	17	73	2	79
	8	91	25	65	1	71
	9	92	27	63	2	68
	10	92	31	60	1	65
	11	90	26	62	2	69
	12	90	26	63	1	70
	13	90	31	58	1	64
	14	89	23	65	1	73
	15	90	32	57	1	63
	16	90	33	55	2	61
	17	89	35	53	1	60
	18	90	36	53	1	59
	19	90	28	60	2	67
	20	90	30	58	2	64
	21	90	38	51	1	57
	22	90	29	60	1	67
	23	88	28	59	1	67
	24	84	28	55	1	65
	25	83	32	49	2	59
	26	83	27	54	2	65
	27	82	26	55	1	67
	28	83	24	57	2	69
	29	81	26	53	2	65
	30	81	28	50	3	62

cCa, corrected calcium

	Week	n	Final evocalcet dosages: 1-2 mg/day			
			Low (n)	Achieved (n)	High (n)	Achievement rate (%)
P	0	131	1	82	48	63
	1	130	5	91	34	70
	2	130	5	96	29	74
	3	127	3	96	28	76
	4	127	5	96	26	76
	5	124	5	92	27	74
	6	124	7	87	30	70
	7	121	6	88	27	73
	8	119	6	94	19	79
	9	117	6	90	21	77
	10	116	7	87	22	75
	11	115	13	78	24	68
	12	112	16	78	18	70
	13	111	8	78	25	70
	14	110	11	68	31	62
	15	108	10	77	21	71
	16	106	12	72	22	68
	17	106	10	72	24	68
	18	106	12	74	20	70
	19	106	10	75	21	71
	20	105	9	75	21	71
	21	101	7	71	23	70
	22	102	9	73	20	72
	23	102	5	80	17	78
	24	102	9	75	18	74
	25	101	6	77	18	76
	26	101	6	76	19	75
	27	101	5	77	19	76
	28	101	9	75	17	74
	29	101	7	80	14	79
	30	99	14	63	22	64
	Week	n	Final evocalcet dosages: 3-4 mg/day			

			Low (n)	Achieved (n)	High (n)	Achievement rate (%)
P	0	90	0	55	35	61
	1	88	1	64	23	73
	2	90	2	62	26	69
	3	90	2	60	28	67
	4	89	4	66	19	74
	5	87	1	61	25	70
	6	87	1	68	18	78
	7	88	4	63	21	72
	8	88	2	71	15	81
	9	86	6	61	19	71
	10	85	8	56	21	66
	11	83	6	59	18	71
	12	83	6	59	18	71
	13	81	6	55	20	68
	14	81	7	50	24	62
	15	79	2	62	15	78
	16	78	5	54	19	69
	17	79	4	58	17	73
	18	77	5	57	15	74
	19	78	3	59	16	76
	20	78	5	55	18	71
	21	77	5	58	14	75
	22	78	6	62	10	79
	23	78	4	59	15	76
	24	76	8	54	14	71
	25	76	5	51	20	67
	26	74	6	50	18	68
	27	74	7	53	14	72
	28	73	5	53	15	73
	29	73	3	58	12	79
	30	73	4	53	16	73
	Week	n	Final evocalcet dosages: 5-8 mg/day			
			Low (n)	Achieved (n)	High (n)	Achievement rate (%)

P	0	92	3	56	33	61
	1	92	2	59	31	64
	2	92	4	58	30	63
	3	91	2	64	25	70
	4	92	2	60	30	65
	5	91	3	62	26	68
	6	92	4	63	25	68
	7	92	5	61	26	66
	8	92	6	57	29	62
	9	92	5	60	27	65
	10	92	5	66	21	72
	11	90	5	64	21	71
	12	90	4	70	16	78
	13	90	7	65	18	72
	14	90	10	58	22	64
	15	90	10	66	14	73
	16	90	13	58	19	64
	17	89	8	60	21	67
	18	90	11	63	16	70
	19	90	10	65	15	72
	20	90	14	59	17	66
	21	90	13	63	14	70
	22	90	15	57	18	63
	23	88	13	54	21	61
	24	84	16	50	18	60
	25	83	12	55	16	66
	26	83	8	59	16	71
	27	82	12	55	15	67
	28	83	13	54	16	65
	29	81	10	57	14	70
	30	81	17	51	13	63

P, phosphate
