# Supplementary material for: Predictive factors requiring high-dose evocalcet in hemodialysis patients with secondary hyperparathyroidism
Source: PLoS One. 2022 Dec 13;17(12):e0279078. doi: 10.1371/journal.pone.0279078 (PMC9746983; doi:10.1371/journal.pone.0279078)
Supplement: S3 File — (PDF) [file pone.0279078.s007.pdf]

S3 File. Changes in the mean (SD) evocalcet dose for 30 weeks.

Week	Final evocalcet dosages (mg/day)		
	1-2 (n=131)	3-4 (n=90)	5-8 (n=92)
0-1	1.08 ± 0.27	1.22 ± 0.42	1.45 ± 0.50
1-2	1.08 ± 0.27	1.21 ± 0.44	1.45 ± 0.50
2-3	1.08 ± 0.27	1.20 ± 0.43	1.45 ± 0.50
3-4	1.38 ± 0.52	1.87 ± 0.52	2.30 ± 0.61
4-5	1.39 ± 0.57	1.99 ± 0.53	2.38 ± 0.55
5-6	1.46 ± 0.60	2.02 ± 0.48	2.41 ± 0.54
6-7	1.51 ± 0.67	2.40 ± 0.65	3.02 ± 0.77
7-8	1.49 ± 0.68	2.51 ± 0.64	3.14 ± 0.72
8-9	1.47 ± 0.75	2.60 ± 0.63	3.25 ± 0.66
9-10	1.45 ± 0.74	2.77 ± 0.69	3.71 ± 0.85
10-11	1.48 ± 0.69	2.77 ± 0.89	3.83 ± 0.85
11-12	1.43 ± 0.71	2.86 ± 0.85	3.96 ± 0.84
12-13	1.48 ± 0.66	2.80 ± 0.94	4.31 ± 1.01
13-14	1.51 ± 0.71	2.89 ± 0.93	4.51 ± 0.99
14-15	1.47 ± 0.76	2.99 ± 0.89	4.61 ± 0.96
15-16	1.48 ± 0.78	3.08 ± 0.86	4.93 ± 1.11
16-17	1.43 ± 0.83	3.11 ± 0.80	5.11 ± 1.13
17-18	1.40 ± 0.82	3.19 ± 0.82	5.14 ± 1.25
18-19	1.40 ± 0.85	3.28 ± 0.72	5.48 ± 1.18
19-20	1.42 ± 0.79	3.32 ± 0.76	5.63 ± 1.24
20-21	1.43 ± 0.81	3.36 ± 0.62	5.78 ± 1.27
21-22	1.40 ± 0.84	3.37 ± 0.61	5.84 ± 1.54
22-23	1.45 ± 0.83	3.42 ± 0.63	5.78 ± 1.77
23-24	1.46 ± 0.83	3.44 ± 0.73	5.98 ± 1.57
24-25	1.40 ± 0.80	3.43 ± 0.72	6.07 ± 1.69
25-26	1.32 ± 0.80	3.51 ± 0.55	6.18 ± 1.58
26-27	1.33 ± 0.71	3.41 ± 0.64	6.30 ± 1.40
27-28	1.35 ± 0.68	3.47 ± 0.50	6.31 ± 1.54
28-29	1.32 ± 0.71	3.32 ± 0.85	6.31 ± 1.54
29-30	1.21 ± 0.75	3.31 ± 0.86	6.23 ± 1.68

SD, standard deviation
