# Supplementary material for: Predictive factors requiring high-dose evocalcet in hemodialysis patients with secondary hyperparathyroidism
Source: PLoS One. 2022 Dec 13;17(12):e0279078. doi: 10.1371/journal.pone.0279078 (PMC9746983; doi:10.1371/journal.pone.0279078)
Supplement: Attachment — Submitted filename: Response_to_Reviewers.docx [file pone.0279078.s009.docx]

PONE-D-21-08923

Predictive factors requiring high-dose evocalcet in hemodialysis patients with secondary hyperparathyroidism

**Responses to review comments *Journal Requirements:***

1. Please ensure that your manuscript meets PLOS ONE's style requirements, including those for file naming. The PLOS ONE style templates can be found at 
[https://journals.plos.org/plosone/s/file?id=wjVg/PLOSOne_formatting_sample_main_body.pdf](https://jpn01.safelinks.protection.outlook.com/?url=https%3A%2F%2Fjournals.plos.org%2Fplosone%2Fs%2Ffile%3Fid%3DwjVg%2FPLOSOne_formatting_sample_main_body.pdf&data=04%7C01%7Cshin.tokunaga.jg%40kyowakirin.com%7C5fdaeb768af74de7093c08d9f72b51fb%7C34db9a5d91ba486ebb82d37d2ee1d84c%7C0%7C0%7C637812590861079225%7CUnknown%7CTWFpbGZsb3d8eyJWIjoiMC4wLjAwMDAiLCJQIjoiV2luMzIiLCJBTiI6Ik1haWwiLCJXVCI6Mn0%3D%7C3000&sdata=%2FxNg2XlPWm%2FLb1s4AYhY1lrUPeaQQA86LaEIKZdHIjY%3D&reserved=0) and 
[https://journals.plos.org/plosone/s/file?id=ba62/PLOSOne_formatting_sample_title_authors_affiliations.pdf](https://jpn01.safelinks.protection.outlook.com/?url=https%3A%2F%2Fjournals.plos.org%2Fplosone%2Fs%2Ffile%3Fid%3Dba62%2FPLOSOne_formatting_sample_title_authors_affiliations.pdf&data=04%7C01%7Cshin.tokunaga.jg%40kyowakirin.com%7C5fdaeb768af74de7093c08d9f72b51fb%7C34db9a5d91ba486ebb82d37d2ee1d84c%7C0%7C0%7C637812590861079225%7CUnknown%7CTWFpbGZsb3d8eyJWIjoiMC4wLjAwMDAiLCJQIjoiV2luMzIiLCJBTiI6Ik1haWwiLCJXVCI6Mn0%3D%7C3000&sdata=eCOfuJkzsCqjSfjF7NIgwBCwQb2iXFa%2FeEREVyU2wNU%3D&reserved=0)

Response: We have confirmed that our manuscript is formatted according to the PLOS ONE style requirements.

2. Thank you for stating the following in the Competing Interests section: "MT received consulting fees from KKC and Ono Pharmaceutical, and lecture fees from KKC, Chugai Pharmaceutical, Bayer, Kissei Pharmaceutical, Torii Pharmaceutical, Fuso Pharmaceutical, and Ono Pharmaceutical. ST, SA, and YE are employees of KKC. NK received lecture fees and travel grants for research meetings from KKC, which are not connected to this research. MF received consulting fees from KKC and Ono Pharmaceutical, lecture fees from KKC, Bayer, Torii Pharmaceutical and Ono Pharmaceutical, and grants from KKC and Bayer. TA received consulting fees from KKC, Astellas Pharma, Bayer, Fuso Pharmaceutical, Japan Tobacco, Ono Pharmaceutical, Sanwa Chemical, Otsuka, GSK, and NIPRO, and lecture fees from KKC, Chugai Pharmaceutical, Bayer, Kissei Pharmaceutical, Torii Pharmaceutical, and Ono Pharmaceutical."

We note that you received funding from a commercial source: Kyowa Kirin Co., Ltd, Ono Pharmaceutical, Chugai Pharmaceutical, Bayer, Kissei Pharmaceutical, Torii Pharmaceutical, and Fuso Pharmaceutical

Please provide an amended Competing Interests Statement that explicitly states this commercial funder, along with any other relevant declarations relating to employment, consultancy, patents, products in development, marketed products, etc.

Within this Competing Interests Statement, please confirm that this does not alter your adherence to all PLOS ONE policies on sharing data and materials by including the following statement: "This does not alter our adherence to PLOS ONE policies on sharing data and materials.” (as detailed online in our guide for authors [http://journals.plos.org/plosone/s/competing-interests](https://jpn01.safelinks.protection.outlook.com/?url=http%3A%2F%2Fjournals.plos.org%2Fplosone%2Fs%2Fcompeting-interests&data=04%7C01%7Cshin.tokunaga.jg%40kyowakirin.com%7C5fdaeb768af74de7093c08d9f72b51fb%7C34db9a5d91ba486ebb82d37d2ee1d84c%7C0%7C0%7C637812590861079225%7CUnknown%7CTWFpbGZsb3d8eyJWIjoiMC4wLjAwMDAiLCJQIjoiV2luMzIiLCJBTiI6Ik1haWwiLCJXVCI6Mn0%3D%7C3000&sdata=ja2o5xSZfEJWWRYx8Pw4YupP%2FeFjIiVs1W%2BeDxfwPaU%3D&reserved=0)).  If there are restrictions on sharing of data and/or materials, please state these. Please note that we cannot proceed with consideration of your article until this information has been declared.

Please include your amended Competing Interests Statement within your cover letter. We will change the online submission form on your behalf.

Response: The sentence “This does not alter our adherence to PLOS ONE policies on sharing data and materials.” has been added after the COI statement in the second paragraph in the cover letter.

3. In your Data Availability statement, you have not specified where the minimal data set underlying the results described in your manuscript can be found. PLOS defines a study's minimal data set as the underlying data used to reach the conclusions drawn in the manuscript and any additional data required to replicate the reported study findings in their entirety. All PLOS journals require that the minimal data set be made fully available. For more information about our data policy, please see [http://journals.plos.org/plosone/s/data-availability](https://jpn01.safelinks.protection.outlook.com/?url=http%3A%2F%2Fjournals.plos.org%2Fplosone%2Fs%2Fdata-availability&data=04%7C01%7Cshin.tokunaga.jg%40kyowakirin.com%7C5fdaeb768af74de7093c08d9f72b51fb%7C34db9a5d91ba486ebb82d37d2ee1d84c%7C0%7C0%7C637812590861079225%7CUnknown%7CTWFpbGZsb3d8eyJWIjoiMC4wLjAwMDAiLCJQIjoiV2luMzIiLCJBTiI6Ik1haWwiLCJXVCI6Mn0%3D%7C3000&sdata=CTWVL6Aibwzk7lLMG9QZ96%2Fm%2Fn%2BV9X5LuEFU25HlA%2BY%3D&reserved=0).

Upon re-submitting your revised manuscript, please upload your study’s minimal underlying data set as either Supporting Information files or to a stable, public repository and include the relevant URLs, DOIs, or accession numbers within your revised cover letter. For a list of acceptable repositories, please see [http://journals.plos.org/plosone/s/data-availability#loc-recommended-repositories](https://jpn01.safelinks.protection.outlook.com/?url=http%3A%2F%2Fjournals.plos.org%2Fplosone%2Fs%2Fdata-availability%23loc-recommended-repositories&data=04%7C01%7Cshin.tokunaga.jg%40kyowakirin.com%7C5fdaeb768af74de7093c08d9f72b51fb%7C34db9a5d91ba486ebb82d37d2ee1d84c%7C0%7C0%7C637812590861079225%7CUnknown%7CTWFpbGZsb3d8eyJWIjoiMC4wLjAwMDAiLCJQIjoiV2luMzIiLCJBTiI6Ik1haWwiLCJXVCI6Mn0%3D%7C3000&sdata=bnJrYkhS9nwvKeuC9fRf5uBla54CXXVkCMIvbDujw8g%3D&reserved=0). Any potentially identifying patient information must be fully anonymized.

Important: If there are ethical or legal restrictions to sharing your data publicly, please explain these restrictions in detail. Please see our guidelines for more information on what we consider unacceptable restrictions to publicly sharing data: [http://journals.plos.org/plosone/s/data-availability#loc-unacceptable-data-access-restrictions](https://jpn01.safelinks.protection.outlook.com/?url=http%3A%2F%2Fjournals.plos.org%2Fplosone%2Fs%2Fdata-availability%23loc-unacceptable-data-access-restrictions&data=04%7C01%7Cshin.tokunaga.jg%40kyowakirin.com%7C5fdaeb768af74de7093c08d9f72b51fb%7C34db9a5d91ba486ebb82d37d2ee1d84c%7C0%7C0%7C637812590861079225%7CUnknown%7CTWFpbGZsb3d8eyJWIjoiMC4wLjAwMDAiLCJQIjoiV2luMzIiLCJBTiI6Ik1haWwiLCJXVCI6Mn0%3D%7C3000&sdata=l0%2B4hKe92GOfsZFvqfBgjzsJ0QCc9TCqwquvcQQ4Xqg%3D&reserved=0). Note that it is not acceptable for the authors to be the sole named individuals responsible for ensuring data access.

We will update your Data Availability statement to reflect the information you provide in your cover letter.

Response: In accordance with your suggestion, the following three files have been added as Supporting information to provide the underlying data used to obtain the percentage of patients achieving the guideline target range for serum iPTH, corrected Ca, and P levels, and changes in the evocalcet dose in our manuscript.

S1 File. Changes in the mean (SD) evocalcet dose for 30 weeks.

S2 File. Serum iPTH, corrected Ca and P levels in patients with SHPT treated with evocalcet for 30 weeks.

S3 File. The percentage of patients with serum iPTH, corrected Ca and P levels above, within, and below the Japanese Society for Dialysis Therapy target ranges for 30 weeks.

4. Please include your full ethics statement in the ‘Methods’ section of your manuscript file. In your statement, please include the full name of the IRB or ethics committee who approved or waived your study, as well as whether or not you obtained informed written or verbal consent. If consent was waived for your study, please include this information in your statement as well.

Response: We confirm that the full names of the ethics committees have been presented in “S1 Table. List of Institutional Review Boards”, and a full ethics statement including the informed written consent has been included in the Study design section (Page 6, Line 111- Page 7, Line 115).

5. Please remove all personal information, ensure that the data shared are in accordance with participant consent, and re-upload a fully anonymized data set.

Response: We confirm that no personal information has been included in any part of our manuscript, and that any participant information has been handled as anonymized data according to the consent form.

***Reviewers' comments:***
Reviewer's Responses to Questions

*Reviewer #1: General comments:
This is a post-hoc analysis of a phase 3 randomized clinical trial which compared head-to-head the efficacy and safety of oral cinacalcet hydrochloride to evocalcet. The post-hoc analysis included 313 hemodialysis patients and looked at the baseline clinical and biochemical parameters determining the response and the use of higher doses of the oral calcimimetic evocalcet. The authors found that at the end of study, median serum PTH level was higher and achievement ratio of the Japanese Society for Dialysis Therapy target range was lower in the high-dose subgroup (216 pg/mL, 58%, respectively) than other subgroups (low: 149 pg/mL, 79%; medium: 149 pg/mL, 73%, respectively). Younger age, prior cinacalcet use, higher levels of serum PTH, corrected calcium, procollagen type 1 N-terminal propeptide, intact fibroblast growth factor-23, and larger maximum parathyroid gland volume were significantly associated with high dose evocalcet (p < 0.05 in all cases).
The authors should consider the following comments and questions.

Specific comments:
1- Was the calcium concentration in the dialysate kept stable during the study? It would be of interest knowing which was the mean (median) calcium concentration in the dialysate fluid for each one of the patient groups.*
Response: It was prohibited to change the prescribed dialysis conditions, including the calcium concentration in the dialysate until week 30 (or at the time of discontinuation). The mean ± standard deviation (SD) dialysate calcium concentration at baseline (week 0) was 2.78 ± 0.19 mEq/L in the low-dose (1–2 mg), 2.77 ± 0.21 mEq/L in the medium-dose (3–4 mg), and 2.79 ± 0.20 mEq/L in the high-dose range (5–8 mg).

Details of the method used to control the dialysate conditions and data of dialysate calcium concentrations have been added to the “Previous phase 3 head-to-head comparison study” section of the Materials and methods (Page 5, Lines 85-88), Results (Page 9, Lines 169-171), and Table 1 (Page 10).

*2- How was the compliance to cinacalcet and evocalcet evaluated?*
Response: The medication adherence rate was calculated using the following formula:

Adherence rate (%) = 100 × number of days administered as prescribed/total number of prescribed days.

We found a high compliance to the medications according to the adherence rate for evocalcet (mean ± SD: 98.51 ± 6.24%) and cinacalcet (98.91 ± 2.24%). The results have been added to the “Previous phase 3 head-to-head comparison study” section of the Materials and methods (Page 6, Lines 99-100).

*3- Circulating levels of 25OHD3 have been demonstrated to be associated with the response to oral cinacalcet. Did the authors assess circulating levels of 25OD3 in this trial?*

Response: Thank you for providing the information regarding the association between 25-hydroxyvitamin D levels and the response to cinacalcet (Zheng CM, *et al*. Nutrients. 2018;10:196). The circulating levels of 25-hydroxyvitamin D were not measured in the previous phase 3 head-to-head cinacalcet and evocalcet study (Fukagawa, *et al*., Kidney Int. 2018;94:818–825). Therefore, we do not have any data to examine an association between 25-hydroxyvitamin D levels and the response to evocalcet.

*Reviewer #2: Here is a list of specific comments. Note: line and page numbering in reviews and comments is based on ruler applied in Editorial Manager-generated PDF.*

*1. Page 4, lines 59–60: This phrase inferred causality. Because the post-hoc analysis lost the benefit of randomization, I suggest using ‘associate’ instead of using “predict”.*
Response: This has been revised to “which factors are associated with patients who…” on Page 4, Line 62.

*2. Page 6, lines 99–101: I suggest indicating the categorized evocalcet dose at week 28 was the primary exposure of interest.*

Response: In this study, we aimed to identify factors to predict which patients will require a high-dose range of evocalcet for SHPT treatment. To achieve this, we analyzed efficacy and safety, and patients’ baseline characteristics associated with the final dose of evocalcet according to the finally-administered evocalcet dose range using the data set obtained in the previous phase 3 head-to-head, active-controlled, randomized, double-blind, comparison study. As you will find in the study design of the previous phase 3 study below, the last dose adjustment was carried out at week 28.

　

(Fukagawa, et al., Kidney Int. 2018;94:818–825)

Therefore, this study was designed (1) to evaluate the efficacy and safety of evocalcet from week 0 to week 30 according to the evocalcet dose range administered to patients at week 28, and (2) to explore the baseline characteristics (week 0) of patients who required the high-dose evocalcet range in week 28.

(1) The efficacy (serum iPTH, cCa, and P, as well as the percentage of patients achieving the JSDT guidelines target ranges) and safety from week 0, when the treatment with evocalcet was initiated, until the end of the study, were evaluated according to the evocalcet dose administered to patients at week 28.

(2) Baseline characteristics of patients who required a final high-dose range of evocalcet during the dose adjustment period, despite a starting low dose of 1 or 2 mg evocalcet, were identified, so that a group of patients who might require a high-dose range of evocalcet could be predicted.

Therefore, the evocalcet dose administered to patients at week 28 was used to stratify the data, and the baseline of each subgroup was set at week 0 when the administration of evocalcet was initiated.

*3. Page 7, line 110: Because the primary exposure of interest was determined at week 28, I suggest clarifying in the manuscript that the baseline for endpoints was week 28.*

Response: As you will find in our response to comment #2, the baseline for the evaluation of endpoints was set at week 0 (start of treatment), not week 28.

*4. Page 7, lines 110–111: I suggest not mentioning “in each subgroup stratified by evocalcet dose range” in the Endpoints section.*

Response: The stratification of the data according to the three ranges of daily evocalcet dose at week 28 was described on Page 6, Lines 108-110 in the Study design section. Therefore, “in each subgroup stratified by evocalcet dose range” has been deleted, accordingly.

*5. Page 7, lines 111–112: This sentence was also irrelevant in the Endpoints section.*

Response: Indeed, it is irrelevant to describe the analysis in the Endpoints section. Therefore, the sentence “Risk factors associated with an evocalcet dose increase and trends in the evocalcet dose were also evaluated for each dose range.” has been deleted.

*6. Page 7, lines 122–126: I suggest clarifying which characteristics were collected at week 28. If a characteristic was collected at week 28, I suggest referring the week-28 value as the “baseline” value.*

Response: As explained in our response to comment #2, the baseline data used for the analyses in this study were collected at week 0 when the evocalcet treatment was initiated.

*7. Page 7, line 127: Did ratios refer to percentages? If so, I suggest replacing ratios with percentages.*

Response: As pointed out, the achievement ratio is shown as a percentage in the JSDT guidelines. Therefore, “ratio” has been replaced with “percentage” in the main text and figures.

*8. Page 7, lines 127–128: I suggest removing “and summary statistics were calculated for continuous data” because it was redundant given the following sentence.*

Response: In accordance with your suggestion, “and summary statistics were calculated for continuous data” has been deleted.

*9. Page 7, line 129: Please write ‘were calculated for continuous data’.*

Response: This has been revised to “were calculated for continuous data” on Page 8, Line 139.

*10. Page 8, line 131: Following Comment #3 above, I suggest limiting to the time points after week 28.*

Response: As you will find in our response to comment #2, the baseline was set at week 0 (start of the treatment), not week 28, for all subgroups. Therefore, trends in the iPTH, cCa, and P levels were analyzed from week 0 to week 30.

*11. Page 8, line 133: Following Comment #3 above, I suggest starting from week 28.*

Response: In response to comment 2, we explained that the baseline of subgroups was not week 28 but week 0 when the treatment with evocalcet was initiated. Therefore, trends in the percentages of patients who achieved JSDT target ranges of iPTH, cCa, and P levels were analyzed from week 0 to week 30.

*12. Page 8, lines 136–140: I did not understand the rationale of this analysis. The evocalcet categories were previously defined and described as the primary exposure of interest. However, the multinomial logistic used the evocalcet categories as the outcome without any justification.*

Response: As we responded to comment 2, the study was designed to evaluate the efficacy and safety of evocalcet from week 0 to week 30 according to the evocalcet dose range that patients received at week 28, and to identify the baseline characteristics (week 0) of patients who required the high-dose evocalcet range at week 28.

*13. Page 8, lines 141–143: Similarly, the safety endpoints and follow-up time should start from week 28 to week 30.*

Response: As we mentioned in our response to comment #12, the study was designed to evaluate the evocalcet dose-dependent (as of week 28) efficacy and safety during week 0 to week 30, and identify patients’ baseline characteristics associated with high-dose evocalcet required at week 28.

*14. Page 8, lines 144–146: I suggest relocating this sentence to the end of the second paragraph of this section.*

Response: As pointed out, the sentence “Statistical differences for categorical variables were estimated using the χ^2^ test (two-tailed), and continuous variables were assessed using the Kruskal–Wallis test and analysis of variance (two-tailed) according to the distribution.” has been moved to the end of the second paragraph of the “Statistical methods” section.

*15. Page 8, lines 150–151: I suggest including a figure depicting the patient flow from the randomization to the safety and full analysis sets.*

Response: The data set used in this study was reported in the previous phase 3 head-to-head, active-controlled, randomized, double-blind, comparison study, which is cited in the Materials and methods and Results sections of our manuscript. The patient flow is shown below for your information (Fukagawa, et al., Kidney Int. 2018;94:818–825).

6. PLOS authors have the option to publish the peer review history of their article ([what does this mean?](https://jpn01.safelinks.protection.outlook.com/?url=https%3A%2F%2Fjournals.plos.org%2Fplosone%2Fs%2Feditorial-and-peer-review-process%23loc-peer-review-history&data=04%7C01%7Cshin.tokunaga.jg%40kyowakirin.com%7C5fdaeb768af74de7093c08d9f72b51fb%7C34db9a5d91ba486ebb82d37d2ee1d84c%7C0%7C0%7C637812590861079225%7CUnknown%7CTWFpbGZsb3d8eyJWIjoiMC4wLjAwMDAiLCJQIjoiV2luMzIiLCJBTiI6Ik1haWwiLCJXVCI6Mn0%3D%7C3000&sdata=4zynxrwa9%2BSxmCqkKwHiomtr918Jzw6N0Xtn8QsbWNs%3D&reserved=0)). If published, this will include your full peer review and any attached files.


If you choose “no”, your identity will remain anonymous but your review may still be made public.


**Do you want your identity to be public for this peer review?** For information about this choice, including consent withdrawal, please see our [Privacy Policy](https://jpn01.safelinks.protection.outlook.com/?url=https%3A%2F%2Fwww.plos.org%2Fprivacy-policy&data=04%7C01%7Cshin.tokunaga.jg%40kyowakirin.com%7C5fdaeb768af74de7093c08d9f72b51fb%7C34db9a5d91ba486ebb82d37d2ee1d84c%7C0%7C0%7C637812590861235432%7CUnknown%7CTWFpbGZsb3d8eyJWIjoiMC4wLjAwMDAiLCJQIjoiV2luMzIiLCJBTiI6Ik1haWwiLCJXVCI6Mn0%3D%7C3000&sdata=ipvdcBsVxVrAnDPkDavUakk76mNu11qBKaC6dmqK86E%3D&reserved=0).

Reviewer #1: No

Reviewer #2: No
