# Supplementary material for: Predictive factors requiring high-dose evocalcet in hemodialysis patients with secondary hyperparathyroidism
Source: PLoS One. 2022 Dec 13;17(12):e0279078. doi: 10.1371/journal.pone.0279078 (PMC9746983; doi:10.1371/journal.pone.0279078)
Supplement: Attachment — Submitted filename: Point-by-point_Response_to_Reviewers.docx [file pone.0279078.s010.docx]

**Reviewer #1:**

**They have properly answered to my main questions. The manuscript has been significantly improved. I do not have further comments.**

**Reviewer #2:** 
**1. Page 6, lines 103–106: I suggest reserving ‘this study’ for the post-hoc analysis and referring the previous phase 3 head-to-head comparison study as ‘****the parent study’.**

*Response*: Accordingly, “the previous phase 3 head-to-head comparison study” has been replaced with “the parent study.”

**2. Page 6, line 104:
(2a) My apologies for misinterpreting the role of the evocalcet categories in Comment #2 of the previous review.
(2b) There were two roles of the evocalcet categories in this study: (1) as an outcome in identifying factors associated with the evocalcet categories; and (2) as a stratification factor in evaluating efficacy and safety of evocalcet.**

**(2c) For the second role, stratifying patients based on a post-baseline factor was likely to introduce bias. In this case, the evocalcet categories was not determined until Week 28 whereas the factors that were evaluated were measured at baseline, Week 0. I suggest considering a more careful analytic plan.**

**(2d) To avoid the aforementioned bias, you may treat Week 28 as baseline. This means that only efficacy and safety endpoints that were measured and collected after Week 28 will be treated as outcomes. Everything collected at or prior to Week 28 could be consider as baseline factor. In this case, the stratified analysis would be OK as long as the outcomes were collected after Week 28.**

*Response*: We understand and appreciate the reviewer’s comments. In accordance with these comments, the analytical plan has been changed and additional analyses have been carried out.

The efficacy and safety analysis plan has been changed to focus on the outcomes in the evaluation period from week 28 to week 30 with baseline at week 28. Accordingly, the efficacy and safety results from week 28 to week 30, as well as patient characteristics in week 28, have been re-analyzed, and the Materials and methods and the Results sections have been revised. The time courses of serum iPTH and corrected Ca levels from week 0 to week 28 are also included as a reference in Figures 2 and 3 to better understand the efficacy of evocalcet during the dose adjustment period.

**3. Page 7, line 115: These endpoints were the outcomes for the second role of the evocalcet categories (see Comment #2b above). For the first role, the outcome would be the evocalcet categories. I suggest explicitly defining the outcome for the first role.**

*Response*: In the section “Analysis for pre-treatment predictive factors associated with final evocalcet dosages,” the sentence “Patient characteristics at week 0 associated with medium and/or high final evocalcet dose ranges were retrospectively identified by a logistic regression model” has been added to define the outcome for the first role (Lines 148-149, Page 9).

**4. Page 7, lines 124–127:
(4a) Comment #15 of the previous review was meant to clarify the full analysis set and the safety analysis set in this study. The figure in the response was for the parent study. I suggest including a patient flow diagram for this study even if it would be a copy of the left-hand side of the original figure.**

*Response*: Accordingly, the patient flow diagram has been added to the manuscript as Fig. 1.

**(4b) In addition, the full analysis and safety sets were with regard to the second role of the evocalcet categories (see Comment #2b above).**

*Response*: The identification of pre-treatment predictive factors was analyzed in the full analysis set. Therefore, the following sentence has been added to the first paragraph of the Statistical methods section (Lines 155-156, Page 9).

“Pre-treatment predictive factors associated with final evocalcet dosages were evaluated in the full analysis set.”

**5. Page 8, lines 134–136: Given Comment #2c above, these comparisons would not be performed unless there was another stratified variable that was defined at baseline. Otherwise, this sentence could be removed.**

*Response*: To be more specific, these sentences have been changed to “The mean and SD, or median and interquartile range, were calculated for continuous data in all patients as well as in each dose subgroup. The statistical significance of differences in dose subgroups was estimated using the χ^2^ test (two-tailed) for categorical variables and the Kruskal–Wallis test and analysis of variance (two-tailed) according to the distribution for continuous variables” (Lines 164-169, Page 10).

**6. Page 8, lines 143–147: This was only for the first role of the evocalcet categories (see Comment #2b above). The analytic plan for the efficacy endpoints were not included.**

*Response*: The analytical plan for the efficacy endpoints has been updated as follows (Lines 170-175, Pages 10). Note that the data from week 0 to week 30 are presented in each figure, but the data from week 28 to week 30 were subjected to the efficacy evaluation.

“For the efficacy endpoints, the median (interquartile range) was obtained for serum iPTH levels, and the mean (SD) was calculated for corrected Ca and P levels from week 28 to week 30 in each subgroup. The ratio of patients who achieved target levels of JSDT for iPTH (60–240 pg/mL), corrected Ca (8.4–10.0 mg/dL), and P (3.5–6.0 mg/dL) [19] were calculated from week 28 to week 30 using those who had levels above, within, and below the target ranges.”
